# Gefäßchirurgische Versorgung im ländlichen Raum – Anpassung an die demografischen und epidemiologischen Erfordernisse

**DOI:** 10.1007/s00772-022-00950-w

**Published:** 2022-11-18

**Authors:** Udo Barth, Dennis Granowski, Martin Lehmann, Frank Meyer

**Affiliations:** 1 Klinik für Allgemein‑, Gefäß- und Viszeralchirurgie, HELIOS-Klinikum Jerichower Land, Burg, Deutschland; 2 Klinik für Allgemein‑, Viszeral‑, Gefäß- und Transplantationschirurgie, Universitätsklinikum Magdeburg A. ö. R., Leipziger Straße 44, 39120 Magdeburg, Deutschland

**Keywords:** Ländlicher Raum, pAVK, Demografischer Wandel, Standortnahe Versorgung, Eingeschränkte Mobilität, Rural areas, PAOD, Demographic change, Local care, Limited mobility

## Abstract

**Einleitung:**

Die demografische Entwicklung in Deutschland und insbesondere in Sachsen-Anhalt (SA) stellt auch die Gefäßchirurgie vor Herausforderungen, denn die Inzidenz der Gefäßerkrankungen ist, dem demografischen Wandel folgend, gestiegen. So wird die Prävalenz der peripheren arteriellen Verschlusskrankheit (pAVK) in den industrialisierten Ländern bei Personen über 60 Jahren bei ca. 10–20 % geschätzt, sodass auch hier mit dem demografischen Wandel die Anzahl der Betroffenen steigen wird. Gerade im ländlichen Raum scheint es für Patienten schwieriger zu sein, eine entsprechende fachärztliche Behandlung zu erreichen.

**Material und Methoden:**

Kompakte narrative Kurzübersicht, basierend auf selektiven Referenzen der aktuellen medizinisch-wissenschaftlichen Literatur und eigenen Erfahrungen aus der täglichen Praxis beim Aufbau einer gefäßchirurgischen Abteilung im ländlichen Raum.

**Ergebnisse:**

Im Jahr 2020 lag die Einwohnerzahl im Landkreis Jerichower Land (SA) bei etwa 89.403 (männlich: 44.489, weiblich: 44.914). Die Altersverteilung in den für die pAVK relevanten Altersgruppen gestaltet sich derart: 65–74 Jahre – gesamt 12,38 %; ab 75 Jahre – gesamt 13,85 %; Durchschnittsalter 48,36 Jahre (Bevölkerungsdichte: 56,4/km^2^). Laut Kassenärztlicher Vereinigung SA kamen 2019 in Burg (SA) 605 Patienten auf einen Arzt.

Insgesamt 5087 Pflegebedürftige gab es 2019 im Landkreis. Bei so einer geringen Bevölkerungsdichte, geringer Arztdichte, hohem Altersdurchschnitt, hohem Anteil an Menschen im Alter von über 75 Jahren sowie einer hohen Anzahl an Pflegebedürftigen ist mit einer Einschränkung der Mobilität und Erreichbarkeit einer gefäßchirurgischen Versorgung zu rechnen, was sich in der hohen Anzahl an pAVK-Stadium IV (FONTAINE) in der initialen Patientenklientel widerspiegelte.

Jeder Aufbau einer gefäßchirurgischen Abteilung ist mit einer erheblichen finanziellen und materiellen Investition verbunden, die der Träger der Einrichtung bereit sein muss vorzunehmen.

Neben der materiellen Investition ist ebenfalls das Vorhandensein eines entsprechend qualifizierten Personals zur Implementierung und Aufrechterhaltung der kontinuierlichen Versorgung zu bedenken.

**Schlussfolgerung:**

Der hohe Anteil von pAVK gefährdeten und daran leidenden Einwohnern in einem ländlich strukturierten Raum mit geringer Bevölkerungs- und Arztdichte erlaubt die Investition in die Neugründung einer gefäßchirurgischen Abteilung, um eine standortnahe Versorgung bei eingeschränkter Mobilität und Selbsthilfe in dieser Patientenklientel zu gewährleisten, damit letztlich aus angemessener gesundheitspolitischer Sicht, aber durchaus auch aus der Perspektive einer relevanten Erlösaussicht.

## Abstract (en)

**Introduction:**

The demographic development in Germany, especially in Saxony-Anhalt (SA), also poses challenges for vascular surgery, as the incidence of vascular diseases has increased following demographic change. For example, the prevalence of peripheral arterial occlusive disease (PAOD) in industrialised countries is estimated at around 10–20% in people over 60 years of age; thus, the number of people affected will also increase here with demographic change. Especially in rural areas, it seems to be more difficult for patients to reach appropriate specialist treatment.

**Material and methods:**

A compact narrative brief review, based on selective references from the current medical-scientific literature and our own experiences from daily practice in setting up a vascular surgery department in a rural area.

**Results:**

In 2020, the population in the rural district of Jerichower Land (SA) was approximately 89,403 (male: 44,489; female: 44,914). The age distribution in the age groups relevant for PAOD is as follows: 65–74 years—total, 12.38%; 75 years and older—total, 13.85%; average age, 48.36 years (population density, 56.4/km^2^). According to the SA Association of Statutory Health Insurance Physicians, there were 605 patients for every doctor in Burg (SA) in 2019.

There was a total of 5087 people in need of long-term care in the district in 2019. With such a low population density, low doctor density, high mean age, high proportion of people over 75 years of age and a high number of people in need of care, limited mobility and accessibility to vascular surgery care are to be expected, which was also reflected in the high number of PAOD of stage IV (FONTAINE) in the initial patient clientele.

Every establishment of a vascular surgery department is associated with a considerable financial and material investment, which the provider of the facility must be prepared to make.

In addition to the material investment, the availability of appropriately qualified staff to implement and maintain continuity of care must also be seriously considered.

**Conclusion:**

The high proportion of residents at risk of and suffering from PAOD in a rural area with low population and doctor density allows investment in the establishment of a new vascular surgery department to ensure local care in this patient group with limited mobility and self-help, thus, ultimately from an appropriate health policy perspective but also from the perspective of a relevant revenue outlook.

## Einleitung

Die demografische Entwicklung in Deutschland und insbesondere in Sachsen-Anhalt (SA) stellt auch die Gefäßchirurgie vor Herausforderungen, denn die Inzidenz der Gefäßerkrankungen ist dem demografischen Wandel folgend gestiegen [1]. So wird die Prävalenz der peripheren arteriellen Verschlusskrankheit (pAVK) in den industrialisierten Ländern bei Personen über 60 Jahren bei ca. 10–20 % geschätzt [2]. Auch das Ulcus cruris zählt mit 57–80 % zu den häufigsten Ursachen für chronische Wunden und zeigte bereits 2012 eine Prävalenz von 0,26–0,64 % auf, sodass auch hier mit dem demografischen Wandel die Anzahl der Betroffenen steigen wird [3]. Wie Rammos et al. in ihrer Studie publizierten, ist die Versorgung von pAVK-Patienten in Deutschland erschreckend mangelhaft. Nur 11 % der Patienten wurden im Jahr 2018 von einem Gefäßchirurgen und nur 8 % von einem Angiologen behandelt. Zudem erhielt nur die Hälfte der Patienten die leitliniengerechte Thrombozytenaggregations- und Statinmedikation [4].

Im Folgenden soll die Frage erörtert werden, ob eine standortnahe gefäßchirurgische Versorgung durch den Aufbau einer neuen gefäßchirurgischen Abteilung in einer ländlichen Region eine sinnvolle Alternative zum Trend der allgemeinen fachspezifischen Zentralisierung bietet.

## Material und Methoden

Kompakte narrative Kurzübersicht, basierend auf selektiven Referenzen der aktuellen medizinisch-wissenschaftlichen Literatur und eigenen Erfahrungen aus der täglichen Praxis beim Aufbau einer gefäßchirurgischen Abteilung im ländlichen Raum.

## Ergebnisse (Eckpunkte)

### Gefäßchirurgische Versorgungssituation in Deutschland

Im Jahr 2019 stellten laut der Grunddaten des Statistischen Bundesamtes 186 gefäßchirurgische Fachabteilungen insgesamt 5529 Betten zur Versorgung. Der Nutzungsgrad betrug insgesamt 72,3 % [5]. Die altersstandardisierte Krankenhausinzidenz von pAVK und arteriellen Embolien/Thrombosen steigerte sich von ca. 190 auf ca. 250 pro 100.000 Einwohner zwischen 2005 und 2018. Dies entsprach einer relativen Zunahme von ca. 33 % [2]. Im gleichen Zeitraum sank die Anzahl der Krankenhäuser von 2139 auf 1925 [5]. Laut den Angaben des Statistischen Bundesamtes ist ein Rückgang gefäßchirurgischer Fachabteilungen von im Jahr 2011 bestehenden 256 Fachabteilungen auf 186 gefäßchirurgische Abteilungen im Jahr 2019 zu verzeichnen [5]. Aus der Ärztestatistik der Bundesärztekammer geht hervor, dass zum 31.12.2020 insgesamt 1418 berufstätige Fachärztinnen und Fachärzte der „Gefäßchirurgie“ beruflich aktiv waren, 1221 davon im stationären Bereich. Zudem gab es 770 berufstätige Personen mit einem Schwerpunkt „SP Gefäßchirurgie“ sowie 34 Personen mit der Bezeichnung „TG Gefäßchirurgie“ (Tab. 1 und 2; [6]). Zur Verbesserung der Qualität der Versorgung gefäßchirurgischer Patienten wurde in den letzten Jahren die Bildung interdisziplinärer Gefäßzentren durch die „Deutsche Gesellschaft für Gefäßchirurgie und Gefäßmedizin“ (DGG) in Kooperation mit der Privaten Akademie der DGG vorangetrieben. Im Januar 2022 waren hier 121 Gefäßzentren aufgelistet [7].

**Table 1:** 

Jahr	Anzahl gefäßchirurgischer Fachabteilungen [*n*]	Aufgestellte Betten [*n*]
*2011*	256	7940
*2019*	186	5529

**Table 2:** 

Gefäßmedizinisch tätige Arztgruppen	Anzahl [*n*]
*Berufstätige FachärztInnen*	
Zum 31.12.2020	1418
Davon im stationären Bereich	1221
*Personen mit einem Schwerpunkt* „SP Gefäßchirurgie“	770
*Personen mit der Bezeichnung* „TG Gefäßchirurgie“	34

### Gefäßchirurgische Versorgungssituation im Jerichower Land

Am 30.09.2021 lag die Einwohnerzahl im Landkreis Jerichower Land (SA) bei etwa 89.276 (männlich: 44.440, weiblich: 44.836). Die Altersverteilung in den für die pAVK relevanten Altersgruppen zum 31.12.2020 gestaltet sich derart: 65–74 Jahre – gesamt 12,38 %; ab 75 Jahre – gesamt 13,85 %; Durchschnittsalter 48,36 Jahre (Bevölkerungsdichte: 56,4/km^2^) [8]. Im Jahr 2019 kamen 605 Einwohner auf eine Ärztin/einen Arzt bzw. Zahnärztin/Zahnarzt im ambulanten Bereich [9]. Insgesamt 5087 Pflegebedürftige gab es 2019 im Landkreis (Tab. 3; [10]). Die Kreisstadt Burg des Jerichower Landes verfügt über ein Krankenhaus der Grund- und Regelversorgung. Eine gefäßchirurgische Fachabteilung bestand bis 2021 nicht. Die nächstgelegenen Krankenhäuser mit einer Gefäßchirurgie befinden sich außerhalb der Kreisgrenzen im Westen mit den Kliniken der Landeshauptstadt Magdeburg (Universitätsklinikum Magdeburg A. ö. R., Klinikum Magdeburg gGmbH, Pfeiffersche Stiftungen Magdeburg), im Süden mit einem Krankenhaus der Grund- und Regelversorgung (AMEOS Klinikum Schönebeck), mit einem Schwerpunktklinikum im Norden (Johanniter-Krankenhaus Genthin-Stendal GmbH) und einem Schwerpunktklinikum im angrenzenden Bundesland Brandenburg (Universitätsklinikum Brandenburg an der Havel). Nach Auskunft der Kassenärztlichen Vereinigung praktizieren im Jerichower Land ein Gefäßchirurg und ein Phlebologe. Es wurden 3 Genehmigungen für die Dopplersonographie extremitätenversorgender Gefäße nach Ziffer 33061 im Landkreis erteilt, 3 Genehmigungen für diabetologisch verantwortliche Ärzte und 11 Genehmigungen zur Behandlung des diabetischen Fußes.

**Table 3:** 

Parameter	Wert
*Einwohnerzahl am 30.09.2021*	89.276
Davon männlich	44.440
Davon weiblich	44.836
*Durchschnittsalter in Jahren*	48,36
*Bevölkerungsdichte in Einwohnern/km*^*2*^	56,40
*Für die pAVK relevante Altersgruppen zum 31.12.2020 in %*	
65–74 Jahre	12,38
Ab 75 Jahre	13,85
*Anzahl der Einwohner auf eine Ärztin/einen Arzt bzw. Zahnärztin oder Zahnarzt 2019 im ambulanten Bereich*	605
*Anzahl der Pflegebedürftigen 2019 im Landkreis*	5087

### Argumente für den Aufbau einer Gefäßchirurgie im Landkreis Jerichower Land

Die Prävalenz der pAVK wird in der Literatur bis auf 10 % geschätzt, wobei davon bis 3 % der Patienten an einer kritischen Extremitätenischämie leiden. Umgerechnet auf die Einwohnerzahl des Jerichower Landes handelt sich um ungefähr 9000 Patienten mit einer pAVK und ca. 270 Patienten mit einer kritischen Extremitätenischämie, die einer dringenden gefäßchirurgischen Behandlung bedürfen, da der Verlust der Extremität der betroffenen Patienten droht. Da die Patienten zudem an weiteren vergesellschafteten Erkrankungen wie Diabetes, koronare Herzkrankheit, Schlaganfällen und Immobilität leiden, ist eine heimatnahe Anbindung zur schnellen und effektiven Versorgung notwendig. Des Weiteren ist die Erkrankung des diabetischen Fußsyndroms zu erwähnen. Jeder vierte Diabetiker erleidet im Laufe seines Lebens ein diabetisches Fußsyndrom. Die Prävalenz des diabetischen Fußulkus liegt bei ca. 2 bis 10 % der Diabetespatienten in Deutschland. Hier ist vor allem die zeitgerechte und kontinuierliche Behandlung wichtig, um die Amputationsrate so gering wie möglich zu halten. Neben der fachgerechten Wund‑, Skelett- und Umfeldbehandlung ist häufig eine gefäßchirurgische Revaskularisation notwendig. Die nicht selten begleitende Niereninsuffizienz der Patienten mit hoher Dialysewahrscheinlichkeit erfordert gerade die Notwendigkeit einer fachgerechten gefäßchirurgischen Behandlung der Shunt- und Vorhofkatheterproblematik. Die chronisch-venöse Insuffizienz aufgrund einer Varicosis ist ein häufiges Krankheitsbild und ist insbesondere im Stadium III nach Widmer mit z. T. ausgedehnten Ulzera der Unterschenkel vergesellschaftet und führt bei den Patienten zu langwierigen und teuren Behandlungen. Eine heimatortnahe umfassende Versorgung (konservativ und chirurgisch) ist für die zum Teil stark immobilisierten Patienten eine Voraussetzung für eine schnelle Heilung und somit Reduktion der Kosten.

### Argumente gegen den Aufbau einer neuen Gefäßchirurgie im Landkreis Jerichower Land

Die Krankenhauslandschaft in Deutschland wird gerade einem Strukturwandel unterzogen mit dem Ziel, die Anzahl der Krankenhausbetten abzubauen, die Aufgaben zwischen den Krankenhäusern neu bzw. besser zu verteilen und die Behandlungsqualität zu erhöhen [11]. Mit dem Gesetz zur Reform der Strukturen der Krankenhausversorgung vom 01.01.2016 will der Gesetzgeber eine qualitätsorientierende Umgestaltung der Krankenhauslandschaft erwirken. Mithilfe der zu publizierenden gesetzlich strukturierten Qualitätsberichte sollen einzelne der Qualitätsprüfungsergebnisse als planungsrelevante Qualitätsindikatoren verwendet werden und bei negativen Ergebnissen eine Schließung von Fachabteilungen durch die Bundesländer möglich machen. Zusammenfassend werden eine Zentralisierung von Krankenhausleistungen, bessere Ressourcenverteilung und ein positiver Einfluss durch die Erfahrung des Personals auf die Behandlungsqualität erwartet [12]. Hier wird zudem argumentiert, dass die kleineren Häuser weder medizintechnisch noch adäquat personell ausgestattet sind und so eine Versorgung der Patienten mit Fachärzten verspätet stattfinden würde, was sich anhand der hohen Zahl an Verlegungen beweisen ließe. Als weiteres Argument wird außerdem die Abhängigkeit der Qualität von der jeweils behandelten Fallzahl („volume-outcome“ Zusammenhang) angeführt [13]. Insofern entspricht das Bestreben der Neuinstallation einer Gefäßchirurgie in der Helios Klinik Jerichower Land in Burg dem Bestreben nach Strukturveränderungen durch den Gesetzgeber nicht ganz.

### Standortvoraussetzungen

Die Helios Klinik Jerichower Land in der Kreisstadt Burg ist ein Krankenhaus der Basisversorgung. Hier werden pro Jahr ca. 11.500 stationäre und ca. 18.000 ambulante Patienten behandelt. Das Krankenhaus verfügt über 231 aufgestellte Betten. Die Klinik ist seit mehreren Jahren akademisches Lehrkrankenhaus der Universität Magdeburg. Zur radiologisch-technischen Grundausstattung gehören ein modernes Computertomogramm und ein Kernspintomograph. Das Angebot für die Patienten umfasst 10 Hauptabteilungen (Innere Medizin, Kardiologie, Allgemein‑, Gefäß- und Viszeralchirurgie, Unfallchirurgie und Orthopädie, Gynäkologie und Geburtshilfe, Anästhesie und Intensivmedizin, Kinder- und Jugendmedizin, plastische und ästhetische Chirurgie, Radiologie, Rückenklinik). Die Innere Medizin ist weiter spezifiziert in Gastroenterologie, Geriatrie und Palliativmedizin. Eine private Dialysepraxis ist im Haus ebenfalls integriert. Die Klinik für Allgemein‑, Gefäß- und Viszeralchirurgie verfügt über 38 Betten, die von 13 examinierten Pflegekräften, 2 Krankenpflegehelfern und 1 Stationsassistentin versorgt werden. Im Arbeitsbereich Allgemein- und Viszeralchirurgie sind neben dem Chefarzt, 3 Oberärzte, 1 Facharzt und 3 Assistenzärzte tätig. Der Arbeitsbereich Gefäßchirurgie wurde mit 2 leitenden Fachärzten für Gefäßchirurgie im Kollegialsystem gegründet. Eine Gefäßassistentin und ein Facharzt für Viszeralchirurgie mit Weiterbildung zum Facharzt für Gefäßchirurgie konnten im Verlauf hinzugewonnen werden.

### Aufbau des Arbeitsbereiches Gefäßchirurgie

**Figure 1:**
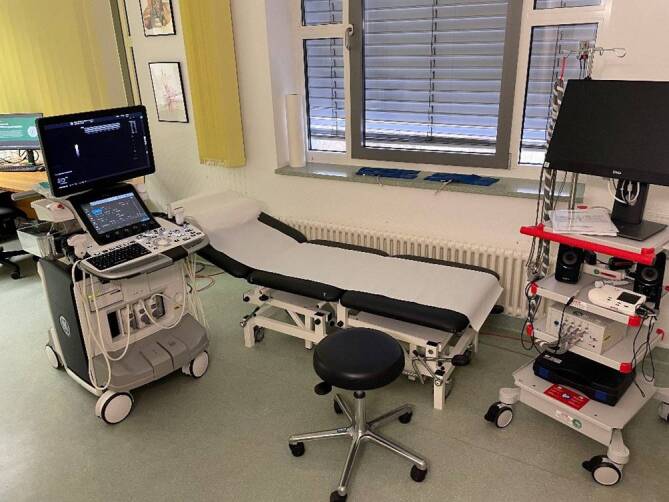

Die Gefäßchirurgie in der Helios Klinik Jerichower Land wurde als integrativer Bestandteil der Klinik für Allgemein- und Viszeralchirurgie geplant, gestaltet und letztlich implementiert. Hintergrund war die gemeinsame Nutzung der personellen, operativ-technischen, räumlichen und strukturellen Gegebenheiten im Sinne einer effektiven Ressourcennutzung, um das Fachgebiet Gefäßchirurgie schnell am Standort zu integrieren. Zudem sollen Synergieeffekte in der pflegerischen Patientenbetreuung und fachübergreifenden Ausbildung der Assistenzärzte genutzt werden. Hierzu wurde die Visite gefäßchirurgischer Patienten in die allgemeine chirurgische Visite integriert. Des Weiteren werden die radiologischen Visiten und Dienstübergaben im Verbund abgehalten. Die kontinuierliche Versorgung gefäßchirurgischer Patienten wird durch ein 24/7-Bereitschaftsdienstsystem gewährleistet. Es erfolgte der Aufbau eines eigenen Diagnostiklabors mit einem Ultraschallgerät und einem Diagnostikmodul zur arteriellen und venösen Gefäßdiagnostik (Abb. 1). In diesem Labor werden zudem 1 × wöchentlich eine Sprechstunde und die erforderliche Gefäßdiagnostik oder -kontrolle von Notfallpatienten und stationären Patienten durchgeführt. Um einen reibungslosen operativen Ablauf zu gewährleisten, wurde ein Flachdetektor-Bildwandler mit Angiographiefunktion installiert und in das PACS(„Picture Archiving and Communication System“)-System integriert. Neben einer Neubeschaffung zahlreicher gefäßchirurgischer Instrumente und Einrichtung spezifischer Instrumentensiebe war der Aufbau eines Lagers mit entsprechenden spezifischen gefäßchirurgischen Materialien (Schleusen, Katheter, Prothesen etc.) notwendig.

Durch die Einstellung einer Operations(OP)schwester mit langjähriger Erfahrung gelang es schnell, das bestehende OP-Personal mit gefäßchirurgischen Operationen und Materialien vertraut zu machen, sodass eine zunehmende Routine beim Umgang mit den operativ-technischen Spezifika eintrat. Im stationären Bereich half den Krankenschwestern die Erstellung spezieller „Standard Operating Procedures“ (SOP), um auf die speziellen Problematiken bei der Betreuung, OP-Vorbereitung und postoperativen Versorgung gefäßchirurgischer Patienten aufmerksam zu machen.

Die Akquisition von Patienten gehörte in der ersten Phase zu einem wesentlichen Bestandteil der Arbeit. Zunächst erfolgte die Bekanntmachung der neuen Abteilung in den lokalen Medien sowie der Besuch zahlreicher Hausarztpraxen im Landkreis. Alle Hausarztpraxen im Landkreis erhielten zudem ein Rundschreiben, in dem das Behandlungsspektrum und die Kontaktdaten mitgeteilt wurden. Zudem wurden Flyer erstellt, die das Diagnostik- und Behandlungsprofil der neuen gefäßchirurgischen Abteilung mit entsprechenden Telefonnummern zeigten. Nach Bekanntgabe in den lokalen Printmedien wurden Telefonsprechstunden zu gefäßchirurgischen Themen durchgeführt, von denen rege Gebrauch gemacht wurde. Genutzt wurden auch Fortbildungsveranstaltungen der Hausärzte, um sich gezielt mit gefäßchirurgischen Vorträgen vorzustellen. Eine Ermächtigung der Kassenärztlichen Vereinigung zur Behandlung gefäßchirurgischer Erkrankungen wurde beantragt, aber mit Hinweis darauf, dass eine andere gefäßchirurgische Praxis im Landkreis sowie andere allgemeinchirurgische Praxen den Bedarf decken, abgelehnt.

### Die Patientenversorgung in der Aufbauphase

In den ersten 12 Monaten wurden insgesamt 213 Patienten stationär und 8 Patienten operativ-ambulant behandelt. Das Durchschnittsalter der Patienten betrug 70,38 (Streubreite: 29–100) Jahre. Darunter litten insgesamt 123 Patienten (57,75 %) an einer pAVK. Diese verteilten sich auf 30 Patienten im Stadium IIB (24,4 %), 12 Patienten im Stadium III (9,76 %), 66 Patienten im Stadium IV (53,66 %), 11 Patienten mit einer inkompletten Ischämie (8,9 %) und 4 Patienten mit einem chronischen Leriche-Syndrom (3,25 %). Zudem wurden 7 Patienten mit einem reinen diabetischen Fußsyndrom, 6 Patienten mit einer asymptomatischen A.-carotis-interna-Stenose, 6 Patienten mit einer arteriellen Embolie und 9 Patienten mit Shuntverschluss operativ behandelt. Die 8 operativ-ambulant behandelten Patienten wurden an einer Varicosis operiert. Aneurysmatische Erkrankungen fanden sich popliteal (2 Patienten), iliakal (1 Patient), infrarenal-aortal (4 Patienten) und subclavial (1 Patient). Ebenfalls erfolgte die operative Behandlung eines Endoleaks Typ I einer infrarenalen aortalen Endoprothese und eines Endoleaks Typ III sowie eines Endoleaks Typ I bei einer thorakalen Endoprothese. Die übrigen Patienten wurden vorwiegend an Infektionen von Wunden und Amputationsstümpfen sowie zur Dialysevorbereitung mit Implantation von Vorhofkathetern und Neuanlagen von AV-Shunts behandelt. Insgesamt wurden 215 operative Eingriffe durchgeführt, davon waren 26 (19,2 %) Notfalleingriffe. Von den 213 Patienten verstarben 5 Patienten (2,3 %) und 5 Patienten erlitten eine behandlungsbedürftige Wundinfektion (2,3 %).

## Diskussion

Mit Installation der Gefäßchirurgie im Klinikum Jerichower Land in Burg wurde ein umfassender Querschnitt an gefäßchirurgischen Therapien angeboten, die im Detail auch über das Niveau eines Krankenhauses der Grund- und Regelversorgung hinausgehen. Von Krankenhausträgerseite wurde zunächst eine Kosten- und Erlösprognose durchgeführt, die sich am Zuweiserverhalten und den Zahlen adäquater Einrichtungen mit einer Gefäßchirurgie orientierte. Die hier ermittelten Zahlen bestätigten die Attraktivität des Aufbaus einer Gefäßchirurgie, sodass das Konzept in die Tat umgesetzt werden konnte.

Mit kontinuierlichem Anstieg der Patientenzahlen stiegen auch die erwirtschafteten CM-Punkte und Erlöse, wobei im Herbst 2021 die Folgen der vierten COVID-19-Welle deutlich zu spüren waren und es wieder zu einem Rückgang der Fallzahlen kam. Erschwerend kam hinzu, dass die erhoffte Ermächtigung zur ambulanten kassenärztlichen Versorgung verweigert wurde, obwohl gerade die fachspezifische Nachbehandlung und Verlaufskontrolle der Patienten mit einer diskontinuierlich verlaufenden pAVK enorm wichtig ist. Die von der Kassenärztlichen Vereinigung erbrachte Begründung, dass die fachspezifische gefäßchirurgische Versorgung der Patienten mit den vorhandenen ambulant praktizierenden Gefäßchirurgen, Phlebologen, Ärzten mit Genehmigung zur Dopplersonographie-Untersuchung und Genehmigung zur Behandlung des diabetischen Fußes ausreichend gesichert wäre, lässt sich insofern nicht nachvollziehen, da der Anteil an Patienten mit einer pAVK im Stadium IV (drohender Extremitätenverlust) in der kurzen Aufbauphase bereits einen Anteil von etwas mehr als der Hälfte der Patienten mit einer pAVK ausmachte. Patienten mit einem Stadium IIB nach Fontaine wurden nur zu 30,4 % stationär behandelt. Dieses Verhältnis war im Jahr 2009 in Deutschland noch umgekehrt, da 31,9 % der Patienten im Stadium III und IV stationär und 68,8 % im Stadium II stationär therapiert wurden [14]. Im Weiteren bestätigt diese erste Analyse der hiesigen pAVK-Patienten die bereits eingangs erwähnte unzureichende ambulante Versorgung gefäßerkrankter Patienten.

Zudem ist die Versorgungssituation im ländlichen Raum als angespannt zu bezeichnen. Zunehmende Defizite in der Infrastruktur, massive Abwanderung junger Leute und der Ärztemangel führen zur Schließung von Krankenhäusern mit dem Ergebnis, dass die zunehmend alternde Bevölkerung mit eingeschränkter Mobilität weite Wege auf sich nehmen muss [15]. Dieses Problem zeigte sich besonders eklatant im Verlauf der COVID-19-Pandemie. Der für die elektive stationäre Aufnahme notwendige negative PCR-Test führte häufig zu mehr Fahrten für die Patienten ins Krankenhaus, zusätzlich zu den üblichen vor- und nachstationären Behandlungen.

Vor diesem Hintergrund ist es wichtig, in Zukunft eine Netzwerkstruktur im Landkreis zu etablieren. Hier hat sich insbesondere bundesweit die „integrierte Versorgung (IV-Struktur)“ bewährt. Der Gesetzgeber hat mit dem § 140a des Sozialgesetzbuches die Möglichkeit geschaffen, dass die Krankenkassen Verträge mit den in Absatz 3 genannten Leistungserbringern über eine besondere Versorgung abschließen können, die eine leistungssektorenübergreifende oder interdisziplinär fachübergreifende Versorgung sowie besondere Versorgungsaufträge unter Beteiligung der Leistungserbringer oder deren Gemeinschaften ermöglichen [16]. Durch die IV-Struktur wird zwar für den Patienten die freie Arzt- und Klinikwahl durch geplante und vertraglich geregelte Abläufe eingeschränkt, jedoch wird eine klare Prozessstruktur und Patientensteuerung geschaffen, sodass der niedergelassene Arzt, das Krankenhaus, die Rehabilitationseinrichtungen und die Krankenkassen koordiniert und partnerschaftlich zusammenarbeiten können [17]. Es bietet sich daher an, den Kontakt zu den diabetologisch verantwortlichen Ärztinnen und Ärzten mit Genehmigung zur Behandlung des diabetischen Fußes aufzunehmen.

Die Aufrechterhaltung eines kontinuierlichen Bereitschaftsdienstsystems über 24 h an 7 Tagen der Woche bereitet mit zwei Fachärzten auf Dauer Schwierigkeiten. Neben erheblichen persönlichen Einschränkungen sind hier auch die Bedingungen des Arbeit-Zeit-Gesetzes im Detail zu beachten. Für die Weiterbildung zum Facharzt für Gefäßchirurgie konnte ein Mitarbeiter der Klinik gewonnen werden. Diese wird jedoch mindestens 3 Jahre (bei vorhandenem Facharzt für Viszeralchirurgie) in Anspruch nehmen, sodass Interimslösungen zu suchen sind. Das Zurückgreifen auf Honorarärzte ist in der Tat schwierig, da neben horrenden Honorarforderungen Probleme in einer schnellen strukturellen Klinikintegration zu sehen sind. So ist über die Etablierung eines Kooperationsvertrages mit einem Maximalversorger/Schwerpunktversorger nachzudenken. Dafür sprechen die Möglichkeiten einer partnerschaftlichen und koordinierten Zusammenarbeit der Kliniken, um eine schnelle und qualitativ hochwertige Versorgung gefäßchirurgischer Patienten zu gewährleisten. Dem gegenüber stehen politische und konkurrierende Interessen als auch die bei den Maximalversorgern vorhandenen personellen und kapazitiven Probleme. Gerade diese strukturellen Probleme der Kliniken in Sachsen-Anhalt sollten Anlass dazu geben, interessenspolitische Hürden zu überwinden, um auch in der Zukunft die anhaltend hohen gefäßchirurgischen Patientenzahlen in guter Qualität zu bewältigen.

Die Integration der Gefäßchirurgie in die Allgemein- und Viszeralchirurgie ist durch Vor- und Nachteile gekennzeichnet. Die Spezialisierung und Zentralisierung gefäßchirurgischer Expertise ist im Hinblick auf die Qualität, Effektivität, Ökonomie und Personalsituation zum vorherrschenden Bestreben geworden. Jedoch gerade im ländlichen Bereich erlaubt die gefäßchirurgische Mitversorgung im Rahmen einer allgemeinchirurgischen Einheit eine bessere ortsnahe Rundumversorgung der Bevölkerung unter der Voraussetzung, dass die durchgeführten Spezialeingriffe in ausreichender Häufigkeit mit der entsprechenden Qualität durch die Gefäßchirurgen durchgeführt werden. Hierbei muss sich die Qualität unbedingt an Qualitätsstandards spezialisierter Einrichtungen messen lassen [18]. So sind z. B. für die Versorgung nichtrupturierter Bauchaortenaneurysmen in der Qualitätssicherungs-Richtlinie zum Bauchaortenaneurysma (QBAA-RL) strukturelle Anforderungen an Einrichtungen in Bezug auf die stationäre Versorgung von Patientinnen und Patienten mit einem Bauchaortenaneurysma notwendig. Erk et al. konnten in einer bundesweiten Studie zeigen, dass es nur geringe Unterschiede der Krankenhausinzidenz nichtrupturierter Bauchaortenaneurysmen und deren Versorgungsform in den vier verschiedenen Kreistypen (Kreistyp 1: kreisfreie Großstadt, Kreistyp 2: städtischer Kreis, Kreistyp 3: ländlicher Kreis mit Verdichtungsansätzen, Kreistyp 4: dünn besiedelter ländlicher Kreis) in Deutschland gibt und sich kein signifikanter Zusammenhang zwischen dem Kreistyp und der Krankenhausmortalität und somit kein Stadt-Land-Gefälle zeigte [19].

In Sachsen-Anhalt gibt es 23 gefäßchirurgische Versorgungseinheiten, wovon 12 als integrativer Bestandteil einer Allgemein- und Viszeralchirurgie und 11 als eigenständige Klinik ausgewiesen sind. Dies unterstreicht, dass die Gefäßchirurgie in Sachsen-Anhalt noch nicht die gebührende Aufmerksamkeit bezüglich ihrer Versorgungsrelevanz bei steigender Prävalenz der Gefäßerkrankungen und Bedeutung für die Versorgung in der Bevölkerung erhält. Als Gründe dafür sind die ökonomischen Zwänge der Kliniken in der strukturschwachen Regionen Sachsen-Anhalt sowie der eklatante Mangel an Ausbildungsassistenten und Fachärzten in der Region zu sehen, der die Nutzung von Synergieeffekten innerhalb der integrierten Struktur erfordert. Jedoch müssen bei vorliegender Struktur Entscheidungen und Qualitätskriterien gegenüber fachfremden Instanzen gerechtfertigt werden. Auch die Ressourcenzuteilung folgt Paradigmen, die die fachliche Qualität und die adäquate Ausstattung nicht aus gefäßmedizinischer Sicht, sondern aus anderen Perspektiven priorisieren. Im Hinblick auf die fachliche Weiterbildung und Lehre sowie die daran gekoppelte Akquisition gefäßchirurgischen Nachwuchses ist es als nachteilig anzusehen, dass es an den Universitätskliniken in Sachsen-Anhalt keinen eigenen Lehrstuhl für Gefäßchirurgie gibt. Wie die Autoren bereits in ihrer Publikation zur chirurgischen Lehre anmerkten, ist die Neuetablierung eigenständiger Kliniken zu fördern, um mit eigenen Ressourcen und Formaten die gefäßchirurgische Lehre weiterentwickeln zu können [20].

Der Aufbau einer Struktur zur Ergebnis- und Qualitätskontrolle ist eine dringliche Aufgabe, um die Arbeit im Arbeitsbereich Gefäßchirurgie auch für den Patienten transparent zu machen. Bisherige Bemühungen, eine Datenbank zur Qualitätssicherung zu erstellen, scheiterten an strukturellen, datenrechtlichen und personellen Problemen. Hier sollte der Zeitpunkt der Datenbankerstellung bei noch übersichtlichen Patientenzahlen nicht verpasst werden. Die Todesfälle wurden in den regelmäßigen Morbiditäts- und Mortalitätskonferenzen auf Klinikebene vorgestellt und diskutiert. Die Behandlung der Wundinfektionen wurde wöchentlich mit dem „Antibiotic Steward“ und der Hygieneschwester abgestimmt.

Auch das Problem der Versorgung von Patienten mit chronischen Wunden ist nach wie vor in der Diskussion. Nur knapp 50 % der Patienten mit chronischen Wunden in Deutschland werden phasengerecht mit Wundauflagen behandelt. Nur ein Drittel aller Patienten mit chronischen Wunden, bei denen eine Kompressionstherapie indiziert ist, erhalten diese in suffizienter Weise [21]. Die chronische Wunde hat daher einen bedeutsamen Stellenwert in der eigenen Stationsroutine und hat für die stationäre Gefäßchirurgie unter Erlösaspekten eine herausragende Bedeutung. Aufgrund der Komplexität und notwendigen Kausaltherapie sollte die chronische Wunde durch Gefäßchirurginnen und Gefäßchirurgen behandelt werden, was angesichts der zu geringen Zahl an Fachärzten nicht zu gewährleisten ist [21].

Am deutlichsten lassen sich die regionalen Unterschiede der Gesundheitsversorgung an der Verteilung der Amputationsraten in den Regionen Deutschlands aufarbeiten. Hier bestehen deutliche regionale Unterschiede. Bei einer Untersuchung der jährlichen bundesweiten Fallzahlen für die Jahre 2011 bis 2015 konnte gezeigt werden, dass überwiegend im Osten und Südosten höhere Amputationsraten bestehen. Insbesondere Kreise in Mecklenburg-Vorpommern, Brandenburg, Sachsen, Sachsen-Anhalt, Thüringen und Bayern zeigten eine höhere „Standardized Mortality Ratio“ (SMR) in mehreren Amputationshöhen. Begründet werden können die auffälligen regionalen Unterschiede durch die hohe altersadjustierte Prävalenz des Diabetes mellitus 2015 für Ostdeutschland von 11,5 %, was sich mit den Regionen der Amputationsraten außerhalb des Konfidenzintervalls deckt, aber insbesondere abhängig ist von der Qualität der Versorgungsstrukturen [22]. Unterschiede in der Intensität der vaskulären Versorgung [22] zusätzlich zu der Erkenntnis, dass weiterhin in Deutschland ca. 40 % der Amputationen bei Patienten mit einer kritischen Extremitätenischämie ohne einen zuvor unternommenen Revaskularisationsversuch durchgeführt werden, bestärken die Notwendigkeit einer Verbesserung der gefäßchirurgischen Versorgung ländlicher und strukturschwacher Regionen.

Um den Qualitätsanspruch auch weiter nach außen zu tragen, wird in Zukunft die Möglichkeit der Spezialisierung auf Kernkompetenzen zu beachten sein. Hier sollte die Zertifizierung nach RAL-Gütezeichen, DGG-Wundzentrum oder DDG-Fußzentrum erwogen werden. Während die RAL-Zertifizierung „Arterie-Vene“, bezogen auf klinisch versorgende Einrichtungen, für eine im Aufbau befindliche Abteilung aufgrund der geforderten 800 Behandlungsfälle im Jahr [23] noch nicht erreichbar erscheint, sind die Anforderungen an ein DGG-Fußzentrum bzw. ICW-DGG-Wundzentrum bereits gegeben und sollten zusammen mit der Klinikleitung in personeller und struktureller Hinsicht entwickelt werden.

## Zusammenfassung

Der hohe Anteil von pAVK-gefährdeten und daran leidenden Einwohnern in einem ländlich strukturierten Raum mit geringer Bevölkerungs- und Arztdichte erlaubt die Investition in die Neugründung einer gefäßchirurgischen Abteilung, um eine standortnahe Versorgung bei eingeschränkter Mobilität und Selbsthilfe in dieser Patientenklientel zu gewährleisten durchaus entgegen dem Bestreben des Gesetzgebers, die Gesundheitsleistungen zu zentralisieren. Gerade Patienten mit gefäßmedizinisch assoziierten Erkrankungen bedürfen einer intensiven heimatnahen Versorgung, da weite Wege zur Behandlung der komplexen Erkrankungen die Compliance der Patienten einschränken. Die Integration einer kleineren gefäßchirurgischen Einheit in eine meist bestehende Allgemein- und Viszeralchirurgie eines Basisversorgers erlaubt eine ortsnahe Rundumversorgung der Patienten, vorausgesetzt, dass die Qualität der gefäßchirurgischen Behandlung den Qualitätsstandards gefäßchirurgischer Zentren entspricht. Die statistisch erfassten zu hohen Amputationsraten in Sachsen-Anhalt sind ein Hinweis auf eine gefäßmedizinische Unterversorgung in dem strukturschwachen Bundesland. Der Aufbau einer gefäßchirurgischen Einheit in einem Krankenhaus der Basisversorgung im ländlichen Bereich kann ein Beitrag zur Reduktion der zu hohen Amputationsrate neben der Gewährleistung einer Rund-um-die-Uhr-gefäßchirurgischen (Notfall‑)Versorgung und einem kompetenten fachspezifischen Wundmanagement als Versorgungseckpfeiler bei adäquater Erreichbarkeit und verkürzten Patientenwegen sowie einer angemessenen Erlössituation sein.
